# Methicillin-resistant *staphylococcus aureus* nosocomial infection has a distinct epidemiological position and acts as a marker for overall hospital-acquired infection trends

**DOI:** 10.1038/s41598-022-21300-6

**Published:** 2022-10-11

**Authors:** Noelle I. Samia, Ari Robicsek, Hans Heesterbeek, Lance R. Peterson

**Affiliations:** 1 Department of Statistics and Data Science, Northwestern University, Evanston, IL 60208 USA; 2 Providence Research Network, Providence, Renton, WA 98057 USA; 3 Population Health Sciences, Faculty of Veterinary Medicine, Utrecht University, 3584 CL Utrecht, The Netherlands; 4 Department of Laboratory Medicine and Pathology and Division of Microbiology, and Department of Medicine and Division of Infectious Diseases, NorthShore University HealthSystem, Evanston, IL 60201 USA; 5 Pritzker School of Medicine, University of Chicago, Chicago, IL 60637 USA

**Keywords:** Infectious diseases, Statistics, Epidemiology

## Abstract

An ongoing healthcare debate is whether controlling hospital-acquired infection (HAI) from methicillin-resistant *Staphylococcus aureus* (MRSA) will result in lowering the global HAI rate, or if MRSA will simply be replaced by another pathogen and there will be no change in overall disease burden. With surges in drug-resistant hospital-acquired pathogens during the COVID-19 pandemic, this remains an important issue. Using a dataset of more than 1 million patients in 51 acute care facilities across the USA, and with the aid of a threshold model that models the nonlinearity in outbreaks of diseases, we show that MRSA is additive to the total burden of HAI, with a distinct ‘epidemiological position’, and does not simply replace other microbes causing HAI. Critically, as MRSA is reduced it is not replaced by another pathogen(s) but rather lowers the overall HAI burden. The analysis also shows that control of MRSA is a benchmark for how well all non-*S. aureus* nosocomial infections in the same hospital are prevented. Our results are highly relevant to healthcare epidemiologists and policy makers when assessing the impact of MRSA on hospitalized patients. These findings further stress the major importance of MRSA as a unique cause of nosocomial infections, as well as its pivotal role as a biomarker in demonstrating the measured efficacy (or lack thereof) of an organization’s Infection Control program.

## Introduction

Much effort has been directed at reducing the acquisition of methicillin-resistant *Staphylococcus aureus* (MRSA) disease in healthcare settings^1–6^. Such efforts have been prompted by data suggesting that MRSA disease is associated with substantial morbidity and cost^7–9^, and that the most important risk for MRSA disease is being colonized in the nares with MRSA^10^. During the COVID-19 pandemic, several drug-resistant pathogens, including MRSA, have been recognized as increasing problems during the pandemic^11,12^ . In a recent study, MRSA is identified to be a leader in global deaths as far as antimicrobial resistance is concerned^12^. This indicates a need for ongoing prevention efforts, even during the SARS-CoV-2 epidemic. MRSA accounts for a sufficiently large proportion of hospital-acquired infection (HAI) disease, thus making it “worth” targeting^13–15^. Because of its impact and societal concerns, MRSA is one of a growing range of pathogens (along with organisms such as CRE, *Acinetobacter*, ESBL-producing bacteria, *Klebsiella* and *Clostridium difficile*) that have a visible ‘public health niche,’ in the sense that Infection Control departments are highly tuned to pick up signals of these pathogens, resulting in ongoing surveillance for changes in infection rates, followed by deployment of various control measures when infections rise. We postulate that, for this reason, MRSA may be able to act as an indicator for overall success of HAI control. An open question is whether MRSA also occupies a distinct ‘*epidemiological* position’ in a hospital setting, interpreted here as a unique combination of factors determining transmissibility and persistence. If so, then this would imply that if MRSA were eliminated, new, similarly virulent organisms would not simply replace it as a cause of nosocomial disease. Hence, these conjectures suggest that if MRSA control efforts succeed in reducing MRSA disease, all-cause HAIs are reduced—and reduced to a substantial extent. These arguments could, in the absence of supporting data, be challenged. It has widely been reported that MRSA accounts for a large proportion of total nosocomial *S. aureus* infection in many hospitals^16–18^. Also, there is evidence that in- and outpatient MRSA disease is adding to rather than simply substituting for methicillin-sensitive *Staphylococcus aureus* infection^19^.

In this study, we present and analyze data to support our hypothesis that MRSA has its distinct epidemiological position with respect to other nosocomial infections in the acute care hospital setting. We also hypothesize that, in such nosocomial settings, MRSA acts as an indicator in explaining trend changes in all non-*S. aureus* infections. For this, we provide a quantitative marker and a threshold level that could be used to gauge hospital performance. We aim to demonstrate that MRSA is additive to the total burden of healthcare-associated all-organism disease and that its control significantly reduces HAI. Our goal is to help infection control programs focus on organisms that impact their overall hospital acquired infection issues.

## Methods

### Data

Our national dataset comes from the database of a medical information technology company (MedMined Services, Care Fusion, Inc., Birmingham, AL) and at the time of collection was one of the largest datasets compiled investigating hospital-acquired infections with over one million patients from 51 different hospitals throughout the United States. MedMined Services is a third-party provider that offers proprietary electronic automated data mining surveillance tool to monitor HAIs. The dataset is diverse and comprises both academic and community hospitals. Our study is based on monthly data (August 2005–September 2008) on the number of HAI caused by various bacteria (MRSA, methicillin-susceptible *S. aureus* [MSSA], and non-*S. aureus* [all other organisms]), along with the number of admissions to each of the 51 hospitals. None of the data was collected at the patient level. The data was aggregated by hospital and on a monthly basis over the 36-month study period; and all the hospitals were de-identified before we received the data. The 51 hospitals included in the study represent all hospitals for which the MedMined data was available for the entire 36-month study period. The dataset covers eight regions as identified by MedMined services over the entire country. No further information about the 51 hospitals (such as geographical location, socio-economic status, urbanicity) was provided.

We use the measured system-wide rates of HAI and MRSA from our four hospitals at NorthShore University HealthSystem for the year 2013, to validate the results of the analysis conducted using the MedMined data above. We also used data from the same network of NorthShore University HealthSystem hospitals for the years 2013–2016, comprising rates of nosocomial MRSA blood stream infections, rates of nosocomial vancomycin resistant enterococci (VRE) blood stream infections, and rates of nosocomial multidrug resistant Gram-negative organism blood stream infections in hospital intensive-care units (ICUs).

HAI was identified based on a validated indicator, namely ‘Nosocomial Infection Marker’ (NIM), as previously investigated^20^. The NIM rates are adjusted for comparison between hospitals based on the case-mix index that is specifically calculated for each facility. The sensitivity and specificity of marker analysis is 0.86 [95% confidence interval: (0.76, 0.96)] and 0.984 [95% confidence interval: (0.976, 0.992)] as previously demonstrated^20^.

### Statistical analysis

**Figure 1:**
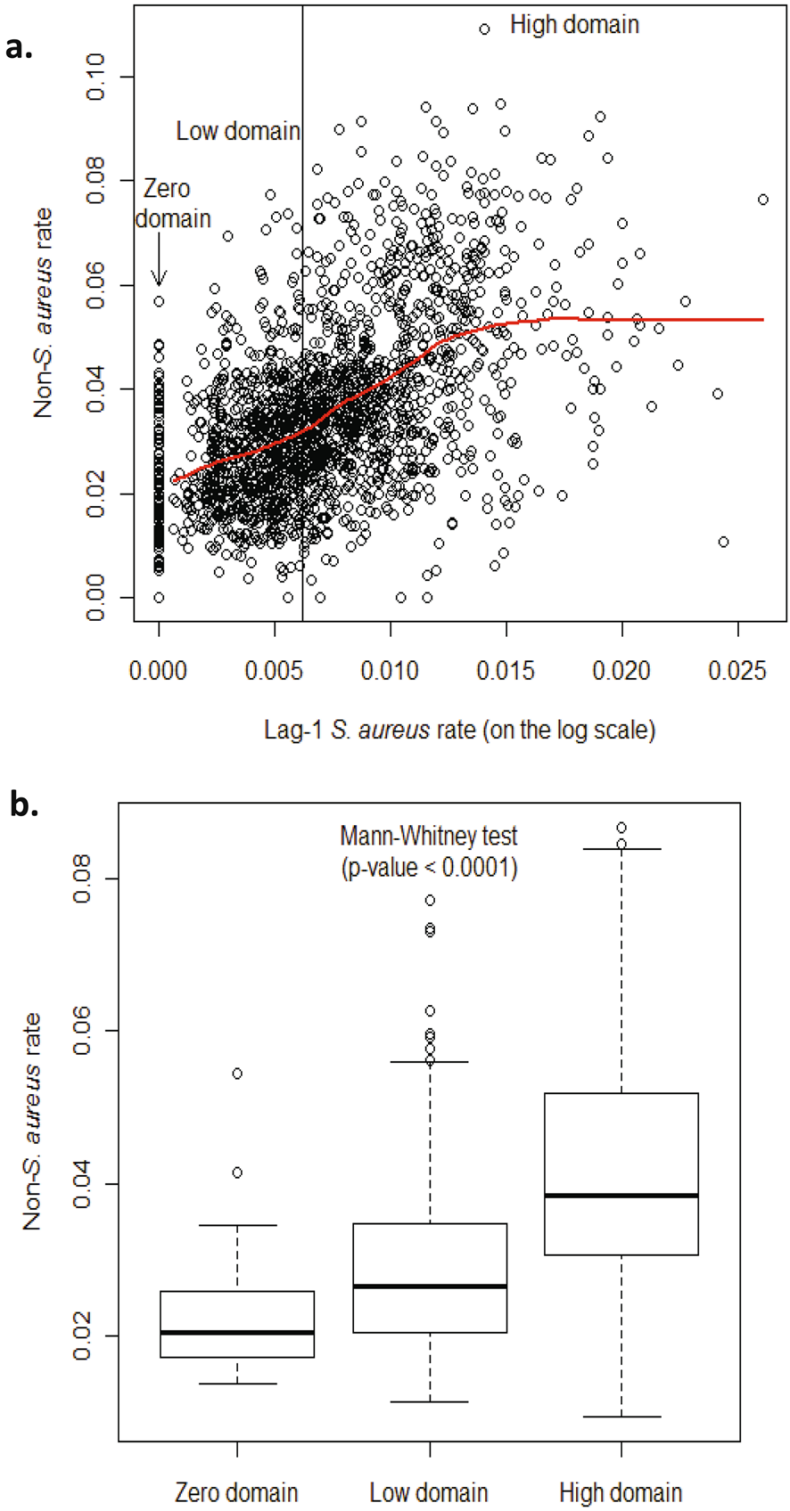
(**a**) Plot of the rate of non-*S. aureus* infections versus the threshold variable, namely the (log-transformed) lag-1 *S. aureus* rate. The zero domain corresponds to a domain such that the lag-1 *S. aureus* rate being equal to zero. The vertical line shows the location of the threshold estimate that defines two non-zero regimes: the low domain and the high domain. The red curve explores nonparametrically the mean of the non-*S. aureus* rate as a function of the lag-1 *S. aureus* rate, by fitting a local regression model. The shape of this red curve attests that the underlying process is nonlinear. (**b**) Boxplots of the fitted non-*S. aureus* rate in each of the 3 domains (zero, low, and high domains). The zero-domain median of the fitted non-*S. aureus* rate is significantly lower than the low-domain median (Mann–Whitney test; *p* value < 0.0001); the low-domain median of the fitted non-*S. aureus* rate is significantly lower than the high-domain median (Mann–Whitney test; *p* value < 0.0001).

We assess the relationship between the number of non-*S. aureus* infections per 1000 admissions (called the rate of non-*S. aureus* infections) and the number of *S. aureus* infections per 1000 admissions (called the rate of non-*S. aureus* infections) and we find it to be nonlinear; see Fig. 1a and the Results section below. As a result of this, we model the nonlinear behavior of the rate of non-*S. aureus* infections explained by changes in the MRSA and MSSA infection rates, using a threshold model for count data known as the Generalized Threshold Model^21^. For this, ${Y}_{t,h}$ denotes the rate of non-*S. aureus* infections in hospital *h* in a given month *t*, and ${A}_{t,h}$ is the number of admissions in hospital *h* in a given month *t*. Then, the number of non-*S. aureus* infections $({Y}_{t,h} {A}_{t,h})$ in hospital *h* at time *t* (measured in months), is assumed to be a Poisson random variable whose mean ${\mu }_{t,h}$=${R}_{t,h} {A}_{t,h}$ is given by

1 $$\log \left( {R_{{t,h}} } \right) = \left\{ \begin{gathered} \alpha _{0} + \alpha _{1} \log (1 + Y_{{t - 1,h}} ) + \alpha _{2} \log (1 + Y_{{t - 2,h}} ),\quad \quad \quad \quad \quad \quad \quad \quad \quad \quad \quad \;if\;Z_{{t - d,h}} = 0 \hfill \\ \beta _{{0,h}} + \beta _{1} X_{{t,h}} + \beta _{2} \log (1 + Y_{{t - 1,h}} ) + \beta _{3} \log (1 + Y_{{t - 2,h}} ) + \beta _{4} \log (1 + Y_{{t - 3,h}} ),~\;if\;0~ < ~Z_{{t - d,h}} \le r \hfill \\ \gamma _{{0,h}} + \gamma _{1} X_{{t,h}} + \gamma _{2} \log (1 + Y_{{t - 1,h}} ) + \gamma _{3} \log (1 + Y_{{t - 2,h}} ) + \gamma _{4} \log (1 + Y_{{t - 3,h}} ),~\quad if\;Z_{{t - d,h}} > r \hfill \\ \end{gathered} \right.$$

where ${Z}_{t-d,h}=\mathrm{log} \left(1+{S}_{t-d,h}\right)$ and ${S}_{t-d,h}$ is the number of *S. aureus* (i.e., MSSA + MRSA) infections per 1000 admissions *d* months earlier in hospital *h* (which we will refer to as the lag-*d S. aureus* rate for that hospital), and ${X}_{t,h}$ is number of MRSA infections per 1000 admissions in hospital *h* in a given month *t* (which we will refer to as the rate of MRSA infections in month *t* for hospital *h*). The parameter $r$ is referred to as the threshold and $d$ is a non-negative integer referred to as the delay or threshold lag, both of which are unknown and being estimated. The variation across hospitals is accounted for in the model through the regression parameters ${\beta }_{0,h}$ and ${\gamma }_{0,h}$ which vary across the 51 hospitals.

We make the following underlying assumptions in our modeling approach. We assume that infections longer than 3 months ago no longer influence the rate of non-*S. aureus* infections. We also assume that hospitals are well-mixed in the sense that every new admission has the same probability to get infected with non-*S. aureus*.

The above fitted model using a Poisson distribution suggests the presence of overdispersion which occurs due to unmeasured factors (e.g., virulence of bacteria) and missing covariates (e.g., number of cultures performed to document infection per hospital and per admission). Hence, we modify the above model to account for this over dispersion by building a hierarchical model such that there is an unobserved random variable ${E}_{t,h}$ having a Gamma($\theta$)/$\theta$ distribution with mean 1 and variance $1/\theta$. Then, conditional on ${E}_{t,h}$, the number of non-*S. aureus* infections $({Y}_{t,h} {A}_{t,h})$ is Poisson distributed with mean ${\mu }_{t,h}{E}_{t,h}$. The marginal distribution of the number of non-*S. aureus* infections ${(Y}_{t,h} {A}_{t,h})$ is then negative binomial with mean ${\mu }_{t,h}$ (with the corresponding infection rate modeled as in Eq. [1] above) and an inflated variance ${\mu }_{t,h}(1+\frac{{\mu }_{t,h}}{\theta })$, where $\theta$ represents the over dispersion parameter (see Venables and Ripley^22^ and McCullagh and Nelder^23^ for a further discussion of modeling using the negative binomial distribution). Using a likelihood-based estimation approach^21^, the threshold delay *d* and the threshold *r* are estimated using a grid search between the 10th percentile and the 90th percentile of the lagged threshold variable ${Z}_{t-d,h}.$

This study was approved by the Institutional Review Board of NorthShore University HealthSystem (Project EH-03–150). As reported in this approval by Institutional Review Board of NorthShore University HealthSystem is the statement 'It is noted that a consent form is not required for this study’. All methods were performed in accordance with the relevant guidelines and regulations.

### Ethics approval

This study was approved by the Institutional Review Board of NorthShore University HealthSystem (Project EH-03–150). As reported in this approval by Institutional Review Board of NorthShore University HealthSystem is the statement ‘It is noted that a consent form is not required for this study’. All methods were performed in accordance with relevant guidelines and regulations.

## Results

**Figure 2:**
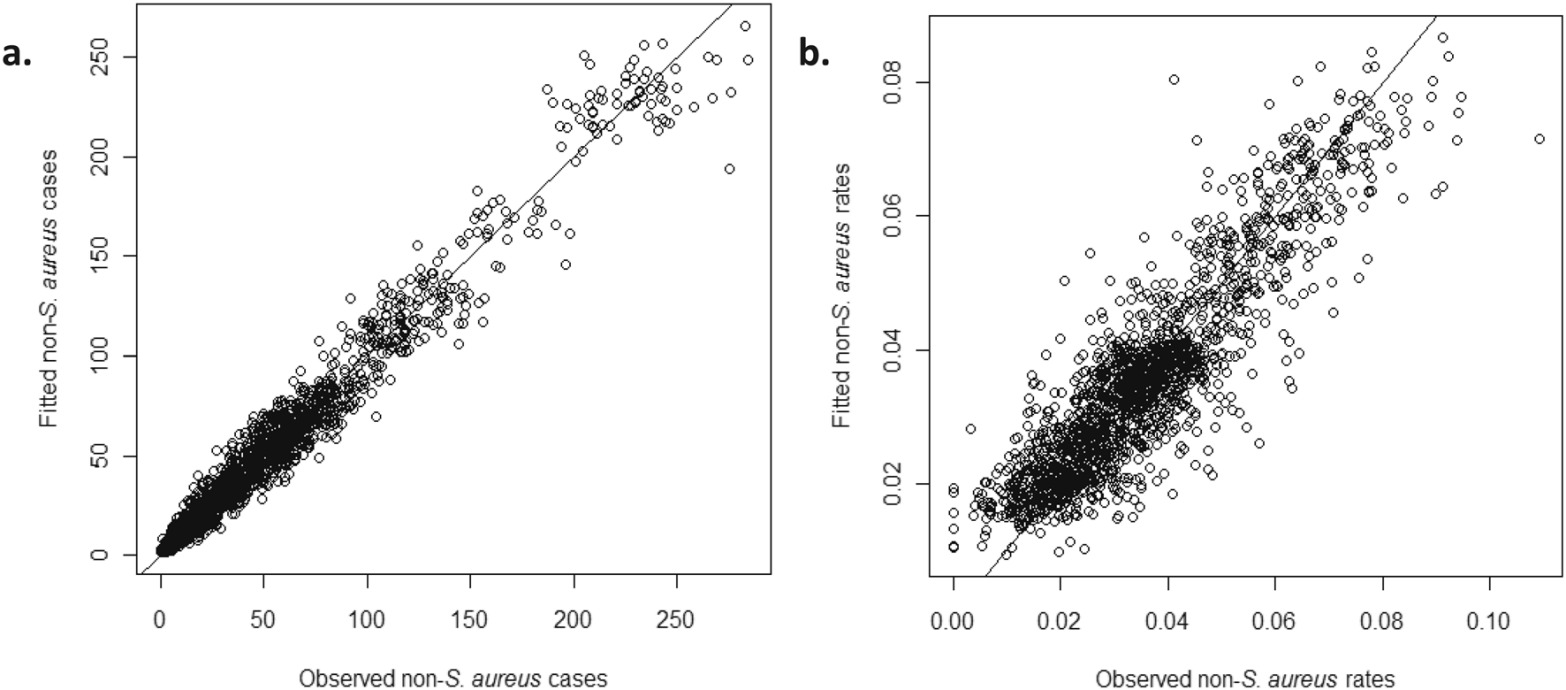
Plot of the fitted values versus the observed values of (**a**) the number of non-*S. aureus* cases and (**b**) the rate of non-*S. aureus* infections.

Figure 1a shows that the rate of non-staphylococcal (i.e., HAI minus MSSA and MRSA) infection varies nonlinearly with the log-transformed *S. aureus* rate of the previous month, i.e., the lag-1 *S. aureus* rate: the strong departure from linearity is unveiled by fitting a nonparametric local regression function (displayed in red in Fig. 1a), confirming that the underlying process is nonlinear. Hence, we model the nonlinear behavior of the rate of non-*S. aureus* infections explained by changes in the MRSA and MSSA rates, using a threshold model for count data^24^, specifically the Generalized Threshold Model^21^, where the threshold variable is the log-transformed lag-1 *S. aureus* rate; see “Methods”. We model the number of non-*S. aureus* infections using a negative binomial distribution that accounts for the over dispersion that occurs due to unmeasured factors (e.g., virulence of bacteria) as well as missing covariates (e.g., microbiological documentation policies)^25,26^; as described in the “Methods”. Table 1 summarizes the maximum likelihood estimates of the parameters in the model given by Eq. [1] in “Methods”. The good agreement between the observed values and the fitted values of the number of non-*S. aureus* cases in Fig. 2a and of the non-*S. aureus* rates in Fig. 2b, attests the credentials and importance of the fitted model in explaining the data; see Supplementary Materials (SM) for details on the model diagnostics.

**Table 1:** Maximum likelihood estimates of the parameters in the fitted threshold model.

Variable	Estimated value	Asymptotic	Asymptotic
		Standard error	95% Confidence interval
**When the lag-1 *S. aureus* rate is zero**			
Intercept ${\alpha }_{0}$	− 4.33	0.098	(− 4.53, − 4.14)
Lag-1 non-*S. aureus* rate ${\alpha }_{1}$	17.7	3.5	(10.7, 24.6)
Lag-2 non-*S. aureus* rate ${\alpha }_{2}$	6.76	3.3	(0.251, 13.2)
**When the lag-1 *S. aureus* rate is positive and ≤ r**			
Hospital-specific intercept ${\upbeta }_{0,\mathrm{h}}$ (See Table S1 in Supplementary Material)			
MRSA rate ${\beta }_{1}$	8.63	4.2	(0.376, 16.8)
Lag-1 non-*S. aureus* rate ${\beta }_{2}$	6.24	1.2	(3.86, 8.62)
Lag-2 non-*S. aureus* rate ${\beta }_{3}$	4.62	1.3	(2.08, 7.16)
Lag-3 non-*S. aureus* rate ${\beta }_{4}$	3.01	1.2	(0.667, 5.35)
**When the lag-1 *S. aureus* rate is positive and > r**			
Hospital-specific intercept ${\upgamma }_{0,\mathrm{h}}$ (See Table S1 in Supplementary Material)			
MRSA rate ${\gamma }_{1}$	10.5	2.4	(5.83, 15.23)
Lag-1 non-*S. aureus* rate ${\gamma }_{2}$	4.47	0.75	(3.00, 5.95)
Lag-2 non-*S. aureus* rate ${\gamma }_{3}$	2.96	0.76	(1.46, 4.45)
Lag-3 non-*S. aureus* rate ${\gamma }_{4}$	2.12	0.77	(0.615, 3.62)

The vertical line in Fig. 1a shows the location of the threshold estimate (0.62%) that defines two non-zero regimes: the low domain and the high domain. As displayed in the box plots of Fig. 1b, the distribution of the fitted non-*S. aureus* rates is shown to be statistically different across the three domains (i.e., the zero, low, and high domains) defined by the lag-1 *S. aureus* threshold variable. In particular, the zero-domain median of the fitted non-*S. aureus* rate is significantly lower than the low-domain median (Mann–Whitney test; *p* value < 0.0001); the low-domain median of the fitted non-*S. aureus* rate is significantly lower than the high-domain median (Mann–Whitney test; *p* value < 0.0001). In addition, Fig. 1b shows that the non-*S. aureus* rate is least variable in the zero domain, with increasing variability from zero to high domains (across the three domains).

The set of explanatory variables used to fit the model in Eq. [1] and their statistical significance in the fitted model reported for each regime in Table 1 show that the trend of total *S. aureus* nosocomial infection of the previous month (i.e., the threshold variable or lag-1 *S. aureus* rate), as well as total non-*S. aureus* nosocomial infection 1, 2, and 3 months earlier, is a benchmark for how well all non-*S. aureus* nosocomial infections are controlled. The lag-1 *S. aureus* rate determines whether or not all non-*S. aureus* nosocomial infections will be moving toward a high or low nosocomial infection domain. These domains are separated by a threshold determined by the lag-1 *S. aureus* rate that indicates whether or not changes in healthcare infection control practices are likely to occur (e.g., practice is likely to change if above the threshold). Below the threshold, the nosocomial infections would appear to be ‘under control’ using current practice; and above the threshold, they would appear ‘out of control’ and require some practice modification or intervention in order to re-enter the low domain. Furthermore, Table 1 shows that the lag-1, -2, and -3 non-*S. aureus* rates are somewhat more dominant in the low nosocomial infection domain (as shown by their parameter estimates and their corresponding 95% CI’s in each domain), where all hospitals with low nosocomial infection rates are tightly clustered as compared to the high domain (which shows more nosocomial infection diversity and variability; see Fig. 1a and b) suggesting that the lagged non-*S. aureus* nosocomial infections are more important in the low domain (along with lag-1 *S. aureus*) and that lag-1 *S. aureus* nosocomial infections are more dominant in the high infection domain. In addition, the fitted model in Eq. [1] (“Methods”) and Table 1 show that the actual rate of MRSA nosocomial infection is a significant predictor of non-*S. aureus* nosocomial infection, and that MRSA is more important in high domain hospitals and may thus dominate all nosocomial infections when these rates are high (Fig. S3 in SM).

Therefore, MRSA at any time was a good marker for identifying the status of overall rate of nosocomial infection at a given healthcare organization. A high overall rate of infection could represent shifts in risk of the underlying population (e.g., changes in use of invasive devices, or changes in performance of certain high-risk procedures, changes in prevalence or ‘colonization pressure’ of certain high-risk pathogens, etc.). Thus, a high rate of MRSA can serve as a trigger to investigate the underlying causes and examine whether or not this increased change in MRSA rate is due to inadequate infection control practice.

The threshold for lag-1 total *S. aureus* nosocomial infection separating the two domains is 0.62% or 6.2 nosocomial *S. aureus* infections per 1000 admissions (see “Methods” for the estimation of the threshold); of these 3.7 to 3.9 are MRSA nosocomial infections per 1000 admissions (mean and median are 0.6 and 0.63 MRSA percentage of *S. aureus*, respectively). In the dataset, the *S. aureus* HAI rate is approximately 17% of the overall HAI rate (mean and median). Thus, one can calculate that the total HAI/*S. aureus* HAI ratio is 5.88. If the threshold variable for separating the domains is 0.62 *S. aureus* infections, then this would suggest that a total HAI rate above 3.65% would serve as a trigger to investigate current infection control practices and possible changes thereof. Thus, hospitals with overall total nosocomial infection proportion less than 3.65% can be classified as ‘Good’ performers in their infection control program, as opposed to being classified as ‘Poor’ performers for having an overall total nosocomial infection proportion above 3.65%. This designation is model informed and is based on the entire realm of hospitals enrolled with MedMined and under consideration in this study. A core component of the model used is its benchmarking capacity that is key to infection prevention and control required to reduce HAI and plays an important role in the absence of national benchmarking designations.

Thus, in summary, the data implies that the MRSA HAI rate can be used as a surrogate to determine how well the overall Infection Control program is performing. If it is ≤ 0.37–0.39%, then the program is likely doing well and is governed by the low domain model where all HAIs are more equal in driving changes in HAI rate, but if it is > 0.39% there is room for improvement (some change in infection control practice may be warranted) and then the total HAI rate is governed by the upper domain model where MRSA disease is most important and likely should be the first target of any intervention.

**Figure 3:**
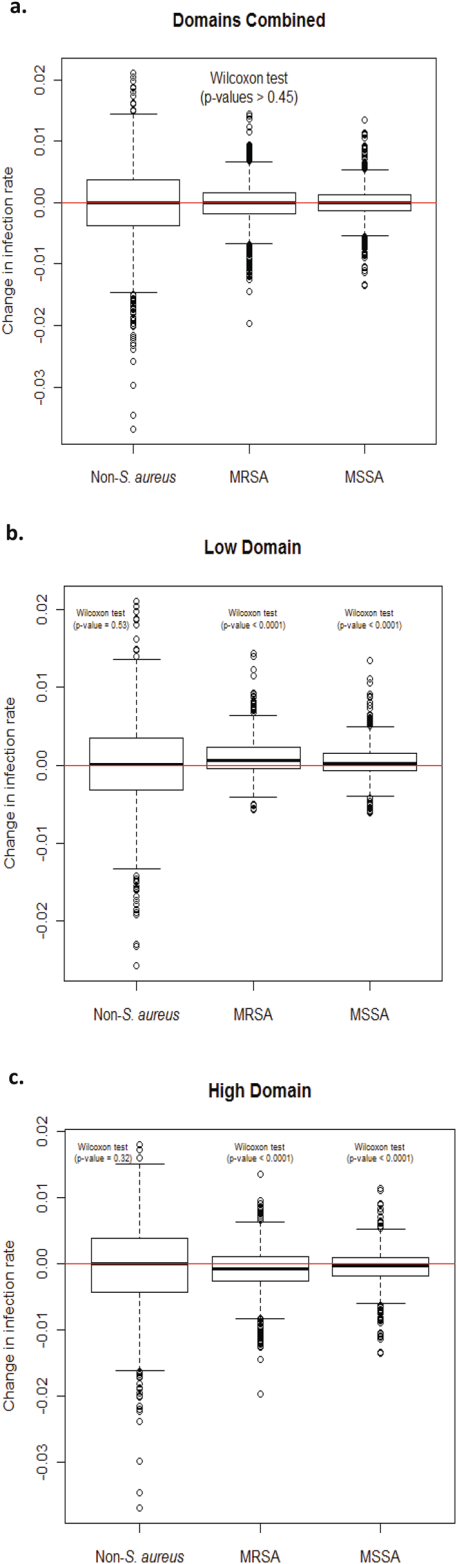
(**a**) Boxplots of the monthly change in the non-*S. aureus* infection rate (i.e., the difference in non-*S. aureus* rate between two consecutive months), the monthly change in the MRSA infection rate, and the monthly change in the MSSA infection rate for all observations corresponding to the *low and high domains*. The red horizontal line refers to a monthly change of zero between two consecutive months. The *medians* of the monthly change in non-*S. aureus* rate, the monthly change in MRSA rate, and the monthly change in MSSA rate are all not statistically different than zero (Wilcoxon test; *p* values > 0.45). (**b**) Boxplots of the monthly change in the non-*S. aureus* infection rate, the monthly change in the MRSA infection rate, and the monthly change in the MSSA infection rate corresponding to the observations in the *low domain* (when lag-1 *S. aureus* rate is less than or equal to 0.62%). The red horizontal line refers to a monthly change of zero between two consecutive months. The *median* change in non-*S. aureus* rate (median = 0.000086) is not statistically different than zero (Wilcoxon test; *p* value = 0.53); however, the *median* change in MRSA rate (median = 0.00054) and the *median* change in MSSA rate (median = 0.00019) are both statistically different than zero (Wilcoxon test; *p* values < 0.0001). (**c**) Boxplots of the monthly change in the non-*S. aureus* infection rate, the monthly change in the MRSA infection rate, and the monthly change in the MSSA infection rate corresponding to the observations in the *high domain* (when lag-1 *S. aureus* rate is greater than 0.62%). The red horizontal line refers to a monthly change of zero between two consecutive months. The *median* change in non-*S. aureus* rate (median =  − 0.000086) is not statistically different than zero (Wilcoxon test; *p* value = 0.32); however, the *median* change in MRSA rate (median =  − 0.00077) and the *median* change in MSSA rate (median =  − 0.00032) are both statistically different than zero (Wilcoxon test; *p* values < 0.0001).

Dividing the data into three domains (zero, low, and high domain) plays a key role in demonstrating that MRSA is additive to nosocomial infections, where movements in all-organism infection rates from one month to the next are not explained by the monthly fluctuations in non-*S. aureus* infection rates, but by the monthly movements in MRSA (and MSSA) infection rates. We studied the behavior of the monthly change of non-*S. aureus* rates (between two consecutive months), the monthly change in MRSA rates, and the monthly change in MSSA rates. Figure 3a shows that by combining all observations from the low and high domains, the distributions of the monthly changes in non-*S. aureus* rates, MRSA rates, and MSSA rates are all centered around zero. Therefore, distinguishing between data from the low and high domains (by modeling this nonlinearity using the threshold model) is required to study the impact of the fluctuations in MRSA rates with infections caused by all organisms. It is only when we separate the data between the low and high domains, that we observe that the monthly change in non-*S. aureus* rates is centered around zero but that the monthly changes in MRSA rates and MSSA rates are distributed around a median that is significantly different than zero (Fig. 3b and c). These findings suggest that MRSA adds to the total burden of all-organism nosocomial infections rather than simply replacing other organisms as a cause of infection.

To validate the estimation of the threshold and the corresponding domains, using data not used in fitting the threshold model for count data, we use our own four-hospital system-wide data at NorthShore University HealthSystem in the year 2013 and we find that the measured system-wide rate of MRSA is 0.15% and the measured system-wide rate of HAI is 2.7%. Table S2 gives the rates of MRSA, VRE, and multidrug-resistant Gram-negative infections, as measured in the network of the NorthShore University HealthSystem for 2013–2016. Further commentary on these findings is included in the Discussion section.

## Discussion

Others have argued that MRSA adds to the total burden of HAI disease in the outbreak setting^27–32^, but we are not aware of any other work that looks at MRSA in general (outbreak and endemic MRSA disease), which rigorously demonstrates using a national dataset that as MRSA goes up so do all HAI and as MRSA goes down so do all HAI. Our results are very relevant to healthcare epidemiologists and policy makers when assessing the impact of MRSA on hospitalized patients. In view of the fact that our study group represents a broad cross section of (community and academic) healthcare facilities, we believe the results are generalizable to most acute care centers in the developed world. First and foremost, we show that MRSA occupies its distinct epidemiological position; hence confronting MRSA on its own as a cause of HAI is worthwhile, as it is unlikely to be replaced by another pathogen, at least in the short term^14^. Additionally, those organizations that succeed in lowering their MRSA rate, should expect the total nosocomial infection rate to decrease, because of the indicator effect of MRSA and its public health niche.

We have tested the threshold hypothesis using our own HAI data for 2013. During that year our 4-hospital, system-wide MRSA rate was 0.15% (lower than the low domain threshold rate of 0.37–0.39%), which would suggest the overall HAI rate being less than 3.65% based on the model; our measured system-wide rate for 2013 was 2.7%. Furthermore, MRSA did not dominate as a pathogen and represented only 5.6% of all organisms causing HAI, again as predicted for an organization in the low domain model. This is also consistent with the model whereby being in the low domain suggests that non-*S. aureus* HAI are more important–in 2013 there was more than 30 microbial species contributing to HAI, with *Escherichia coli* being most common, followed by enterococci, *Clostridium difficile*, yeast, and then *S. aureus*.

Evolutionary speaking, we believe that the MRSA-niche in a hospital setting is also not likely to be filled in the long term by another pathogen since MRSA is an adaptive change in the parent microbe, *S. aureus*, which has always been an aggressive pathogen^33^. When antibiotics became widely available that could suppress or treat *S. aureus*, the organism adapted and became more antibiotic resistant (MRSA)^33,34^. In doing so, it added to or replaced the parent (MSSA) as a hospital pathogen^35^. Our results illustrate that if MRSA is controlled, it will indeed not be replaced at least in the short term. This is corroborated by data from the Netherlands and the Scandinavian countries, which have a (relatively) long history of controlling and monitoring MRSA, and where no ‘new’ organism has stepped up to fill the void for at least 25 years^36,37^. Furthermore, *S. aureus* behaves similarly in that if one controls it (via decolonization) before surgery, the post-operative infection rates fall four to six-fold and *S. aureus* is not replaced by another pathogen^38,39^.

Our study has limitations. Since the cost of the MedMined Services program is not inconsequential, it could be argued that our hospital cohort is willing to dedicate more resources than most to their infection control program and are thus not representative of most U.S. facilities. While this may be true, this weakness is countered by the strength of the data that is completely objective since information is drawn directly from laboratory information systems and electronic admission-discharge-transfer records with no human ‘interpretation’ that could bias the reported HAI results. Furthermore, other large healthcare systems have invested large resources in MRSA control^40^, so we believe our hospital data set is representative of the broad HAI model patterns in U.S. hospitals. The finding that there was a spread of MRSA rates that correlated with overall nosocomial infection trends does not contradict the representativeness of the data we used, despite this potential bias. Since this study was completed, MedMined was acquired by BD integrated analytics and the authors can no longer access additional data. This would not alter the investigation reported but would impair any further data investigation.

Further research of the underlying factors involved in the MRSA epidemiological position is warranted. This, however, would require a different study design. For such a study, one should look at the entire hospital, and its interaction with the outside world (society), as an ecosystem in which pathogens encounter favorable and unfavorable circumstances for their survival, reproduction and spread, and where different pathogens are under different pressure. Likely factors are number of patients (i.e., potential susceptible hosts for the nosocomial pathogen species), turnover rate of patients, intensity of treatment of individual patients, differences in susceptibility among patients (e.g., age-related or related to cause of hospitalization), the level of infection control and hand hygiene measures, compartmentalization of patients (metapopulation structure), and the connectivity of the contact network of patients and healthcare workers, in addition to antibiotic stewardship.

Microbiology laboratory data from hospitals are critical for following the evolution of multidrug resistant organisms^41^. Unfortunately, the hospital laboratory cannot detect potential nosocomial infections that are sometimes not well microbiologically documented (e.g., surgical site infections) or occur after discharge of the patient^41^.

The database used is relatively old (2005–2008). At that time, other pathogens, in particular, extended-spectrum β-lactamase producing *Enterobacteriacae* and carbapenemase-producing *Enterobacteriacae* (CPE), caused fewer nosocomial infections compared to MRSA. While many countries have reported an improvement in MRSA infection rates during these last 10 years, studies have also highlighted the progression of infections caused by multidrug resistant Gram negative organisms^42^. An effective overall infection prevention program is multifaceted including antimicrobial stewardship, interventions targeted on problematic pathogens (such as CPE surveillance), and monitoring of hand hygiene. While data about other emerging pathogens was not available to use in this study, we reviewed four years of data (2013–2016) from the NorthShore University HealthSystem network of four hospitals to give insight into the observed pattern of these other critical pathogens. The rates of the nosocomial blood stream infections caused by MRSA and VRE, and the rates of nosocomial blood stream infections caused by multidrug resistant Gram negative organisms in hospital intensive-care units showed that other infections were not replacing MRSA. Interestingly, VRE and resistant Gram negatives were actually either decreasing (VRE) or simply not emerging (the Gram negatives); see Table S2 in SM, supporting our hypothesis.

In summary, the proposed MRSA indicator in this study should be seen as an important part of a set of indicators. One could argue that the dataset used in this study is very elaborate from a time where a possible signal to be derived from MRSA control is not yet masked by the much larger number of important bacterial threats we have today. We recognize how the world of healthcare associated infections has changed and what this could mean for the use of this MRSA marker. Thus, follow-up investigation should be conducted to include these organisms using a large dataset in contemporary time.

In conclusion, while healthcare-associated MRSA infection has been recognized as an important adverse event, our analysis shows its major importance as a unique cause of nosocomial infection, as well as its pivotal role as a biomarker in demonstrating the measured efficacy (or lack thereof) for a given Infection Control program in the acute care setting. Until current electronic databases that keep track of all HAIs become widely used, our data suggest that to lower the HAI rate, hospitals can benefit from an infection control program that includes monitoring MRSA disease rates and enhances comprehensive patient safety efforts when these rates are rising.

## Supplementary Information

Supplementary Information.

## Data Availability

The datasets generated during and/or analysed during the current study are available from the corresponding author on reasonable request.
