# Supplementary material for: Methicillin-resistant staphylococcus aureus nosocomial infection has a distinct epidemiological position and acts as a marker for overall hospital-acquired infection trends
Source: Sci Rep. 2022 Oct 11;12:17007. doi: 10.1038/s41598-022-21300-6 (PMC9552150; doi:10.1038/s41598-022-21300-6)

**Supplementary Material (SM)**

**Methicillin-Resistant *Staphylococcus aureus* Nosocomial Infection has a Distinct Epidemiological Position and Acts as a Marker for Overall Hospital-Acquired Infection Trends**

Noelle I. Samia^a,1^, Ari Robicsek^b^, Hans Heesterbeek^c^, and Lance R. Peterson^d,e^

^a^Department of Statistics and Data Science, Northwestern University, Evanston, IL 60208, USA

^b^Providence Research Network, Providence, Renton, WA 98057, USA

^c^Population Health Sciences, Faculty of Veterinary Medicine, Utrecht University, 3584 CL, Utrecht, The Netherlands

^d^NorthShore University HealthSystem, Department of Laboratory Medicine and Pathology and Division of Microbiology, Evanston, IL 60201, USA

^e^Pritzker School of Medicine, University of Chicago, Chicago, IL 60637, USA, retired

^1^Corresponding author:

N. I. Samia: phone: +1-847-491-5772; fax: +1-847-491-4939; e-mail: [n-samia@northwestern.edu](mailto:n-samia@northwestern.edu)

**Supplementary Material (SM)**

Methods

Table S1: Maximum likelihood estimates of the hospital-specific intercepts in the two regimes with nonzero *Staphylococcus aureus* (*S. aureus*) rates.

Table S2: Rates of nosocomial MRSA blood stream infections, rates of nosocomial vancomycin resistant enterococci (VRE) blood stream infections, and rates of nosocomial multidrug resistant Gram-negative organism blood stream infections in hospital intensive-care units (ICUs), for the network of NorthShore University HealthSystem hospital for the years 2013-2016.

Figure S1: Plot of the deviance residuals versus the fitted values (on the log scale).

Figure S2: Normal probability plot of the deviance residuals.

Figure S3: Plot of the observed non-*Staphylococcus aureus* (non-*S. aureus*) infection rate versus the observed MRSA infection rate.

**Supplementary Material (SM)**

**Methicillin-Resistant *Staphylococcus aureus* Nosocomial Infection has a Distinct Epidemiological Position and Acts as a Marker for Overall Hospital-Acquired Infection Trends**

**Methods**

*Statistical model diagnostics*. Model diagnostics are used to check the adequacy of the fitted model. In particular, the plot of the deviance residuals versus the fitted values in Fig. S1 does not indicate any significant heteroscedastic pattern. In addition, the normal probability plot of the deviance residuals in Fig. S2 does not show any significant departure from the assumption of normality. The good agreement between the observed values and the fitted values of the number of non-*S. aureus* cases in Fig. 2A (in the main text) and of the non-*S. aureus* rates in Fig. 2B (in the main text), is further evidence to the usefulness and goodness of the fitted model in explaining the data.

**Table S1.** Maximum likelihood estimates of the hospital-specific intercepts in the two regimes with nonzero *Staphylococcus aureus* (*S. aureus*) rates. Note that ‘NA’ indicates that the corresponding hospital does not have any observation in the regime taken into consideration.

| Variable | Estimated  Value | Asymptotic  Standard Error | Asymptotic 95%  Confidence Interval |
| --- | --- | --- | --- |
| **When the lag-1 *S. aureus* rate is positive and ≤ r** | | | |
| Hospital-specific intercept $\beta_{0,h}$ |  |  |  |
| $h=1$  $h1$  $h=1$ | NA | NA | NA |
| $h=2$ | -4.04 | 0.073 | (-4.18, -3.90) |
| $h=3$ | -4.27 | 0.094 | (-4.45, -4.08) |
| $h=4$ | -3.90 | 0.078 | (-4.05, -3.75) |
| $h=5$ | -4.31 | 0.065 | (-4.44, -4.18) |
| $h=6$ | -3.81 | 0.078 | (-3.96, -3.66) |
| $h=7$ | -4.41 | 0.060 | (-4.53, -4.30) |
| $h=8$ | -3.74 | 0.099 | (-3.94, -3.55) |
| $h=9$ | -3.63 | 0.11 | (-3.85, -3.40) |
| $h=10$ | -3.84 | 0.16 | (-4.16, -3.53) |
| $h=11$ | -3.87 | 0.073 | (-4.01, -3.73) |
| $h=12$ | -3.87 | 0.072 | (-4.01, -3.72) |
| $h=13$  $h1$  $h=1$ | -4.01 | 0.063 | (-4.14, -3.89) |
| $h=14$ | -4.21 | 0.069 | (-4.35, -4.08) |
| $h=15$ | -4.23 | 0.18 | (-4.60, -3.90) |
| $h=16$ | -4.37 | 0.16 | (-4.68, -4.07) |
| $h=17$ | -3.93 | 0.086 | (-4.10, -3.76) |
| $h=18$ | -4.18 | 0.046 | (-4.27, -4.09) |
| $h=19$ | -4.00 | 0.083 | (-4.16, -3.84) |
| $h=20$ | NA | NA | NA |
| $h=21$ | -3.75 | 0.16 | (-4.06, -3.45) |
| $h=22$ | -3.88 | 0.088 | (-4.05, -3.71) |
| $h=23$ | -3.37 | 0.18 | (-3.73, -3.02) |
| $h=24$ | -4.12 | 0.063 | (-4.24, -3.99) |
| $h=25$ | -3.69 | 0.093 | (-3.87, -3.51) |
| $h=26$ | -3.83 | 0.072 | (-3.97, -3.69) |
| $h=27$ | -3.88 | 0.073 | (-4.02, -3.74) |
| $h=28$ | -4.32 | 0.13 | (-4.58, -4.06) |
| $h=29$ | -4.22 | 0.054 | (-4.32, -4.11) |
| $h=30$ | -3.99 | 0.092 | (-4.17, -3.81) |
| $h=31$ | -4.28 | 0.050 | (-4.37, -4.18) |
| $h=32$ | -3.77 | 0.086 | (-3.93, -3.60) |
| $h=33$ | -3.71 | 0.10 | (-3.91, -3.50) |
| $h=34$ | -3.98 | 0.080 | (-4.14, -3.83) |
| $h=35$ | -3.91 | 0.18 | (-4.26, -3.56) |
| $h=36$ | -3.83 | 0.11 | (-4.06, -3.61) |
| $h=37$ | -4.04 | 0.093 | (-4.23, -3.86) |
| $h=38$ | -4.67 | 0.36 | (-5.46, -4.03) |
| $h=39$ | -3.80 | 0.095 | (-3.99, -3.61) |
| $h=40$ | -4.16 | 0.063 | (-4.28, -4.03) |
| $h=41$ | -3.96 | 0.065 | (-4.09, -3.84) |
| $h=42$ | -3.83 | 0.080 | (-3.99, -3.67) |
| $h=43$ | NA | NA | NA |
| $h=44$ | -3.76 | 0.081 | (-3.92, -3.60) |
| $h=45$ | -4.12 | 0.060 | (-4.23, -4.00) |
| $h=46$ | -3.97 | 0.083 | (-4.13, -3.80) |
| $h=47$ | -3.82 | 0.089 | (-4.00, -3.65) |
| $h=48$ | -3.75 | 0.12 | (-3.99, -3.50) |
| $h=49$ | -4.20 | 0.11 | (-4.41, -3.99) |
| $h=50$ | -3.89 | 0.064 | (-4.01, -3.76) |
| $h=51$ | -3.79 | 0.20 | (-4.18, -3.41) |
| Variable | Estimated  Value | Asymptotic  Standard Error | Asymptotic 95%  Confidence Interval |
| **When the lag-1 *S. aureus* rate is positive and > r** | | | |
| Hospital-specific intercept $\gamma_{0,h}$ |  |  |  |
| $h=1$  $h1$  $h=1$ | -3.56 | 0.059 | (-3.68, -3.45) |
| $h=2$ | -3.80 | 0.049 | (-3.90, -3.71) |
| $h=3$ | -3.99 | 0.25 | (-4.53, -3.53) |
| $h=4$ | -3.83 | 0.054 | (-3.93, -3.72) |
| $h=5$ | -4.12 | 0.097 | (-4.32, -3.94) |
| $h=6$ | -3.65 | 0.057 | (-3.76, -3.54) |
| $h=7$ | -3.97 | 0.18 | (-4.35, -3.63) |
| $h=8$ | -3.60 | 0.056 | (-3.71, -3.49) |
| $h=9$ | -3.85 | 0.086 | (-4.02, -3.68) |
| $h=10$ | -3.44 | 0.061 | (-3.56, -3.32) |
| $h=11$ | -3.69 | 0.055 | (-3.80, -3.58) |
| $h=12$ | -3.65 | 0.080 | (-3.81, -3.50) |
| $h=13$  $h1$  $h=1$ | -3.97 | 0.11 | (-4.20, -3.75) |
| $h=14$ | -3.93 | 0.063 | (-4.05, -3.81) |
| $h=15$ | -4.74 | 0.28 | (-5.35, -4.24) |
| $h=16$ | -4.26 | 0.15 | (-4.55, -3.98) |
| $h=17$ | -3.68 | 0.046 | (-3.77, -3.59) |
| $h=18$ | -4.13 | 0.065 | (-4.26, -4.01) |
| $h=19$ | -3.92 | 0.046 | (-4.01, -3.83) |
| $h=20$ | -3.38 | 0.073 | (-3.52, -3.24) |
| $h=21$ | -3.39 | 0.075 | (-3.54, -3.25) |
| $h=22$ | -3.70 | 0.075 | (-3.85, -3.56) |
| $h=23$ | -3.46 | 0.073 | (-3.60, -3.32) |
| $h=24$ | -3.83 | 0.062 | (-3.95, -3.71) |
| $h=25$ | -3.54 | 0.059 | (-3.65, -3.42) |
| $h=26$ | -3.70 | 0.052 | (-3.80, -3.60) |
| $h=27$ | -3.68 | 0.050 | (-3.78, -3.59) |
| $h=28$ | -4.01 | 0.047 | (-4.10, -3.92) |
| $h=29$ | -4.22 | 0.086 | (-4.39, -4.05) |
| $h=30$ | -3.65 | 0.048 | (-3.74, -3.56) |
| $h=31$ | -4.09 | 0.12 | (-4.32, -3.86) |
| $h=32$ | -3.71 | 0.048 | (-3.81, -3.62) |
| $h=33$ | -3.83 | 0.076 | (-3.98, -3.68) |
| $h=34$ | -3.77 | 0.095 | (-3.96, -3.59) |
| $h=35$ | -3.39 | 0.069 | (-3.53, -3.25) |
| $h=36$ | -3.68 | 0.049 | (-3.77, -3.58) |
| $h=37$ | -3.81 | 0.082 | (-3.98, -3.66) |
| $h=38$ | -4.40 | 0.25 | (-4.92, -3.95) |
| $h=39$ | -3.59 | 0.078 | (-3.74, -3.44) |
| $h=40$ | -4.10 | 0.10 | (-4.30, -3.91) |
| $h=41$ | -3.73 | 0.053 | (-3.84, -3.63) |
| $h=42$ | -3.70 | 0.054 | (-3.81, -3.60) |
| $h=43$ | -3.47 | 0.061 | (-3.59, -3.35) |
| $h=44$ | -3.67 | 0.050 | (-3.76, -3.57) |
| $h=45$ | -3.98 | 0.080 | (-4.14, -3.82) |
| $h=46$ | -3.90 | 0.049 | (-3.99, -3.80) |
| $h=47$ | -3.64 | 0.048 | (-3.73, -3.55) |
| $h=48$ | -3.62 | 0.063 | (-3.75, -3.50) |
| $h=49$ | -4.08 | 0.086 | (-4.25, -3.91) |
| $h=50$ | -3.73 | 0.069 | (-3.87, -3.60) |
| $h=51$ | -3.37 | 0.079 | (-3.53, -3.22) |

**Table S2.** Rates of nosocomial MRSA blood stream infections, rates of nosocomial vancomycin resistant enterococci (VRE) blood stream infections, and rates of nosocomial multidrug resistant Gram-negative organism blood stream infections in hospital intensive-care units (ICUs), for the network of NorthShore University HealthSystem hospital for the years 2013-2016.

There were approximately 60,000 hospital admissions during 2013 with an annual increase of 5%. There were approximately 225,000 hospital inpatient days during 2013 with an annual increase of 5%. There were approximately 4,400 ICU admissions during 2013 with an annual increase of 5%. There were approximately 17,000 ICU inpatient days during 2013 with an annual increase of 5%. The value <0.02 was chosen as the number where this appears as that is the lower limit of detection for this marker in ICU patients.

| Year | Nosocomial MRSA Blood Stream Infections (Rate per 10,000 patient-days) | Nosocomial VRE Blood Stream Infections (Rate per 10,000 patient-days) | Nosocomial Multidrug Resistant Gram-negative Organism Blood Stream Infections in hospital ICUs (Rate per 10,000 patient-days) |
| --- | --- | --- | --- |
| 2016 | 0.21 | 0 | <0.02 |
| 2015 | 0.33 | 0.05 | <0.02 |
| 2014 | 0.22 | 0.11 | <0.02 |
| 2013 | 0.49 | 0.33 | <0.02 |

**Figure legends**

**Figure S1.** Plot of the deviance residuals versus the fitted values (on the log scale).

**Figure S2.** Normal probability plot of the deviance residuals.

**Figure S3.** Plot of the observed non-*Staphylococcus aureus* (non-*S. aureus*) infection rate versus the observed MRSA infection rate: observations in the low domain (i.e., when the nonzero lag-1 *S. aureus* infection rate is less than or equal to 0.62%) are indicated with open circles; observations in the high domain (i.e., when the nonzero lag-1 *S. aureus* infection rate is strictly greater than 0.62%) are denoted as +. The blue solid curve illustrates the median (i.e., 50^th^ percentile) effect of changes in the MRSA rate on the non-*S. aureus* rate, in the low domain, with the other covariates fixed at their median values. The blue dashed lines refer to the (10^th^ and 90^th^) percentile effect of change in the MRSA rate on the non-*S. aureus* rate, in the low domain, with the other covariates fixed at their 10^th^ and 90^th^ percentiles, respectively. Similarly, the red solid curve illustrates the median (i.e., 50^th^ percentile) effect of changes in the MRSA rate on the non-*S. aureus* rate, in the high domain, with the other covariates fixed at their median values. The red dashed lines refer to the (10^th^ and 90^th^) percentile effect of change in the MRSA rate on the non-*S. aureus* rate, in the high domain, with the other covariates fixed at their 10^th^ and 90^th^ percentiles, respectively.

**Figure S1.**

**
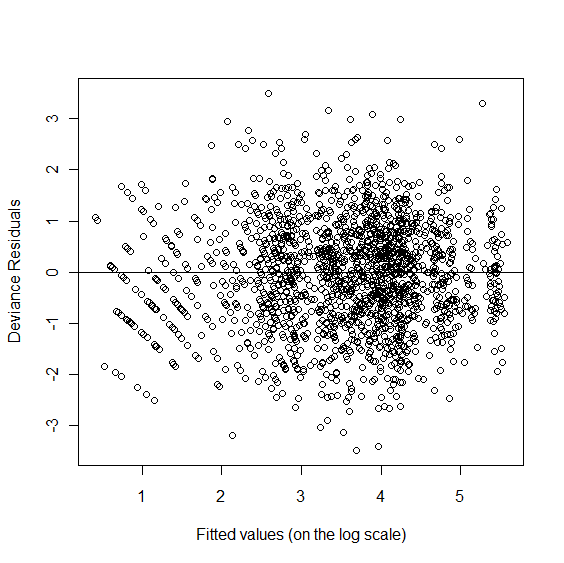
**

**Figure S2.**

**
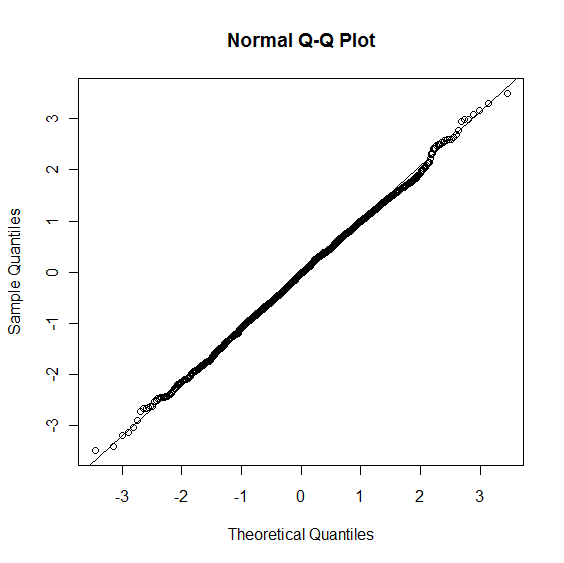
**

**Figure S3.**


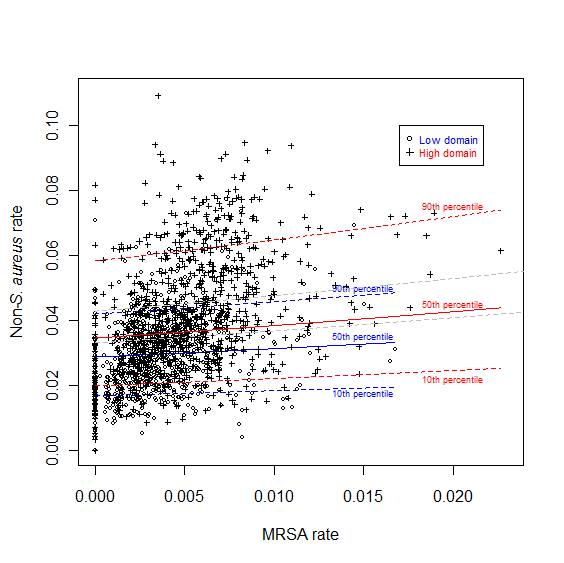

Supplement: Supplementary file 1 — Supplementary Information. [file 41598_2022_21300_MOESM1_ESM.docx]
